# Supplementary material for: The Systems Biology Research Tool: evolvable open-source software
Source: BMC Syst Biol. 2008 Jun 29;2:55. doi: 10.1186/1752-0509-2-55 (PMC2446383; doi:10.1186/1752-0509-2-55)
Supplement: Additional file 1 — SBRT Archive. An archive of the current version of the Systems Biology Research Tool. [file 1752-0509-2-55-S1.zip › sbrt-1.4.0/doc/developers_guide/api/sbrt/kernel/io/package-use.html]

Uses of Package sbrt.kernel.io


|  |  |  |
| --- | --- | --- |
| **Overview** \| **Package** \| Class \| **Use** \| **Tree** \| **Deprecated** \| **Index** \| **Help** | |  |
| PREV   NEXT | **FRAMES**    **NO FRAMES**     **All Classes** |  |


---


## **Uses of Package sbrt.kernel.io**

| Packages that use sbrt.kernel.io | |
| --- | --- |
| **sbrt.kernel** | Provides interfaces to define processes. |
| **sbrt.kernel.fba.proc** | Provides classes and interfaces for executing Flux Balance Analysis processes. |
| **sbrt.kernel.io** | Provides classes and interfaces used to perform kernel-level I/O. |
| **sbrt.kernel.math.algebra.proc** | Provides classes and interfaces for executing algebra-related processes. |
| **sbrt.kernel.math.comb.proc** | Provides classes and interfaces for performing combinatorial analysis-related processes. |
| **sbrt.kernel.math.geom.proc** | Provides classes and interfaces for performing geometry-related processes. |
| **sbrt.kernel.math.graph\_theory.proc** | Provides classes and interfaces for performing graph theoretical processes. |
| **sbrt.kernel.math.stat.proc** | Provides classes and interfaces for performing statistical processes. |
| **sbrt.kernel.utilities.proc** | Provides classes and interfaces for performing utility processes. |
| **sbrt.shell.io** | Provides classes and interfaces for reading and writing files. |
| **sbrt.shell.io.fba** | Provides classes and interfaces for reading and writing files relevant to Flux Balance Analysis. |
| **sbrt.shell.mng** | Provides classes and interfaces for creating process managers. |
| **sbrt.shell.mng.fba** | Provides classes and interfaces for creating Flux Balance Analysis process managers. |

Classes in sbrt.kernel.io used by sbrt.kernel

****ResultsManager****
  
          This interace is used to represent a results-manager.

Classes in sbrt.kernel.io used by sbrt.kernel.fba.proc

****ResultsManager****
  
          This interace is used to represent a results-manager.

Classes in sbrt.kernel.io used by sbrt.kernel.io

****AbstractResultsManager****
  
          This class is a skeleton implemenation of `ResultsManager`.

****ResultsManager****
  
          This interace is used to represent a results-manager.

****WarningHandler****
  
          This interface is used to represent a class of objects that deal with warning
messages.

Classes in sbrt.kernel.io used by sbrt.kernel.math.algebra.proc

****ResultsManager****
  
          This interace is used to represent a results-manager.

Classes in sbrt.kernel.io used by sbrt.kernel.math.comb.proc

****ResultsManager****
  
          This interace is used to represent a results-manager.

Classes in sbrt.kernel.io used by sbrt.kernel.math.geom.proc

****ResultsManager****
  
          This interace is used to represent a results-manager.

Classes in sbrt.kernel.io used by sbrt.kernel.math.graph\_theory.proc

****ResultsManager****
  
          This interace is used to represent a results-manager.

Classes in sbrt.kernel.io used by sbrt.kernel.math.stat.proc

****ResultsManager****
  
          This interace is used to represent a results-manager.

Classes in sbrt.kernel.io used by sbrt.kernel.utilities.proc

****ResultsManager****
  
          This interace is used to represent a results-manager.

Classes in sbrt.kernel.io used by sbrt.shell.io

****ResultsManager****
  
          This interace is used to represent a results-manager.

Classes in sbrt.kernel.io used by sbrt.shell.io.fba

****ResultsManager****
  
          This interace is used to represent a results-manager.

Classes in sbrt.kernel.io used by sbrt.shell.mng

****ResultsManager****
  
          This interace is used to represent a results-manager.

Classes in sbrt.kernel.io used by sbrt.shell.mng.fba

****AbstractResultsManager****
  
          This class is a skeleton implemenation of `ResultsManager`.

****ResultsManager****
  
          This interace is used to represent a results-manager.

---


|  |  |  |
| --- | --- | --- |
| **Overview** \| **Package** \| Class \| **Use** \| **Tree** \| **Deprecated** \| **Index** \| **Help** | |  |
| PREV   NEXT | **FRAMES**    **NO FRAMES**     **All Classes** |  |


---
